# Hydrogen Sulfide Rescues Microglia From HIV Tat‐Driven Ferroptosis: Implications for HIV‐Associated Neuroinflammation

**DOI:** 10.1002/cns.70977

**Published:** 2026-06-12

**Authors:** Aitizaz Ul Ahsan, Frida L. Martínez‐Cuevas, Elias Horanieh, Seema Singh, Uma Maheswari Deshetty, Palsamy Periyasamy, Shilpa Buch

**Affiliations:** ^1^ Department of Pharmacology and Experimental Neuroscience University of Nebraska Medical Center Omaha Nebraska USA

**Keywords:** ferroptosis, microglia, NeuroHIV, neuroinflammation, sodium hydrosulfide

## Abstract

**Aim:**

HIV transactivator of transcription (Tat) protein induces oxidative stress, neuroinflammation, and glial dysfunction in NeuroHIV. Ferroptosis, an iron‐dependent form of regulated cell death driven by lipid peroxidation, has emerged as a contributor to HIV‐associated neurocognitive disorders. This study aimed to determine whether hydrogen sulfide (H_2_S) mitigates HIV Tat‐induced ferroptosis in microglial cells.

**Methods:**

BV2 microglial cells were pretreated with sodium hydrosulfide (NaHS; 100 μM), an H_2_S donor, followed by exposure to recombinant HIV Tat (100 ng/mL, 48 h). Ferroptotic indices, including cytosolic Fe^2+^ accumulation, lipid peroxidation, reactive oxygen species (ROS) generation, and cell membrane damage, were assessed using fluorescence‐based assays and lactate dehydrogenase (LDH) release. Expression of ferroptosis‐related and antioxidant proteins was analyzed by western blotting, and proinflammatory cytokine release was quantified by qPCR.

**Results:**

NaHS pretreatment significantly attenuated HIV Tat‐induced Fe^2+^ accumulation, ROS generation, lipid peroxidation, and LDH release. Mechanistically, NaHS suppressed pro‐ferroptotic mediators, acyl‐CoA synthetase long‐chain family member 4 (ACSL4) and 4‐hydroxynonenal (4‐HNE), while restoring solute carrier family 7‐member 11 (SLC7A11) and glutathione peroxidase 4 (GPX4) expression. NaHS also reduced HIV Tat‐induced IL1β, IL6, and TNFα secretion.

**Conclusion:**

These findings demonstrate that H_2_S protects HIV Tat‐exposed microglia by suppressing ferroptosis and restoring cellular homeostasis. Collectively, these results identify H_2_S signaling as a promising mechanistic target for further investigation in NeuroHIV‐associated neuroinflammation.

## Graphical abstract

HIV Tat induces ferroptosis and proinflammatory activation in microglia through iron accumulation and oxidative stress. Hydrogen sulfide donor NaHS suppresses HIV Tat‐induced ferroptosis by restoring redox balance, reinforcing the SLC7A11‐GPX4 axis, and attenuating microglial inflammation, highlighting hydrogen sulfide as a potential therapeutic strategy for NeuroHIV.

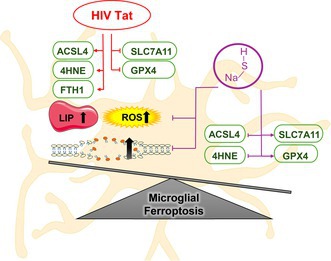

## 1 Introduction

The advent of combination antiretroviral therapy (cART) has transformed human immunodeficiency virus (HIV) infection from a fatal disease into a manageable chronic condition, greatly increasing life expectancy. Currently, approximately 39 million people are living with HIV worldwide [1]. Despite effective viral suppression in peripheral blood by cART, latent viral reservoirs persist in the central nervous system (CNS), driving chronic neuroinflammation and leading to neurological complications collectively termed NeuroHIV [2]. Although cART has markedly reduced the incidence of HIV‐associated dementia to 1%–2%, the prevalence of milder forms of HIV‐associated neurocognitive disorders, however, has increased [3]. The HIV Transactivator of transcription (Tat) protein plays a central role in viral replication and neuroinflammation [4]. The sustained expression of HIV Tat in cerebrospinal fluid (CSF) and postmortem brain tissues of HIV‐infected individuals underscores its pathogenic importance [5]. HIV Tat is actively secreted from infected cells and released into the extracellular milieu, where it can enter neighboring cells via heparan sulfate proteoglycan‐mediated endocytosis [6]. Once internalized, HIV Tat accumulates in endolysosomes, inducing lysosomal deacidification and dysfunction [7].

Within the CNS, HIV primarily enters via infected macrophages and CD4^+^ T cells, which then infect resident microglia, the brain's innate immune cells. These HIV‐infected or HIV Tat‐exposed microglia become hyperactivated, releasing neurotoxins, proinflammatory cytokines, and chemokines, leading to chronic immune activation and neuronal injury [8]. This neuroinflammatory cascade, exacerbated by oxidative stress and mitochondrial dysfunction, contributes significantly to the pathogenesis of NeuroHIV [9]. Together, these observations emphasize the need for anti‐Tat adjunctive therapies to complement cART.

Among the cellular processes implicated in NeuroHIV, iron dysregulation plays a critical role [10]. Iron is essential for neuronal metabolism and microglial function; however, its excess accumulation in microglia promotes proinflammatory activation and neurodegeneration [11, 12]. Excess iron catalyzes Fenton reactions, generating hydroxyl radicals that induce oxidative and lipid damage. This redox imbalance leads to ferroptosis, a distinct, iron‐dependent form of programmed cell death characterized by lipid peroxidation [13]. Mechanistically, ferroptosis represents a balance between pro‐ferroptotic insults, such as acyl‐CoA synthetase long chain family member 4 (ACSL4)‐mediated lipid peroxidation, and anti‐ferroptotic defenses mediated by the solute carrier family 7‐member 11 (SLC7A11/Xct)‐glutathione peroxidase 4 (GPX4) antioxidant system, which detoxifies lipid peroxides using glutathione [14], and disruption of this axis sensitizes cells to ferroptotic death [15].

Emerging studies, including ours, imply ferroptosis in the neuropathogenesis of HIV infection [16, 17]. Our previous work demonstrated that HIV Tat dysregulates microglial ferroptosis in both HIV transgenic rats and postmortem brains of HIV‐positive individuals [16]. Moreover, given the importance of iron in viral replication, iron chelators have been shown to inhibit key stages of the HIV‐1 life cycle [18, 19]. Recent evidence indicates that the endogenous gasotransmitter hydrogen sulfide (H_2_S) is depleted during HIV infection [20], contributing to oxidative stress, inflammatory imbalance, and alterations in its normal physiological signaling functions within the nervous and cardiovascular systems [21, 22]. The effects of H_2_S have been examined using both endogenous H_2_S and exogenous donors, such as sodium hydrosulfide (NaHS), which has demonstrated notable neuroprotective properties, largely through microglial regulation [23, 24]. These protective actions are often attributed to its ability to counter oxidative stress by elevating intracellular cysteine and glutathione, a key antioxidant [25]. Additionally, studies show that NaHS and other H_2_S donors modulate cysteine‐glutamine transporters [26, 27]. H_2_S also directly or indirectly influences the activity of transcription factors such as NF‐κB, which plays a critical role in triggering HIV‐1 reactivation from latency [28, 29]. Collectively, these findings suggest that H_2_S signaling is interconnected with physiological processes relevant to HIV disease progression.

Given the established role of H_2_S in regulating redox balance, mitochondrial bioenergetics, and inflammatory responses, we hypothesized that NaHS could modulate HIV Tat‐induced ferroptosis in microglial cells. To test this, we investigated HIV Tat‐induced ferroptosis in BV2 microglia using approaches previously developed in our laboratory [16]. A combination of biochemical, molecular, and microscopic techniques was employed to elucidate how NaHS mitigates HIV Tat‐induced ferroptotic cell death. This study, for the first time, delineates the protective efficacy of NaHS against HIV Tat‐induced ferroptosis in microglia. Our findings demonstrated that NaHS conferred protection by restoring redox and iron homeostasis, downregulating pro‐ferroptotic markers such as ACSL4, 4‐hydroxynonenal (4‐HNE), reducing labile iron levels, and enhancing GPX4 expression via the SLC7A11‐GPX4 axis, ultimately alleviating the accumulation of reactive oxygen species (ROS). NaHS pretreatment did not significantly impact the expression of FTH1 in HIV Tat‐exposed BV2 microglia. These results establish a protective role for NaHS in restoring homeostasis within HIV Tat‐exposed microglia and suggest that targeting this pathway may represent a viable therapeutic strategy for NeuroHIV‐associated neuroinflammation.

## 2 Materials and Methods

### 2.1 Chemicals and Reagents

Endotoxin‐free, HIV‐1 recombinant Tat (1032‐10) was purchased from ImmunoDX. Sodium hydrosulfide (Cat. No. 161527) and Erastin (Cat. No. E7781) were purchased from Sigma‐Aldrich (St. Louis, MO, USA). BODIPY 581/591 C11 Lipid Peroxidation Sensor (Cat. No. D3861) and the Image‐iT LIVE Green Reactive Oxygen Species Detection Kit (Cat. No. I3600) were obtained from ThermoFisher Scientific Inc. Eugene, Oregon, USA. The Iron Assay Kit (Colorimetric; Cat. No. ab83366), and LDH Assay Kit (Cat. No. ab65393), were purchased from Abcam (Boston, MA, USA) and RSL3 (Cat. No. 19288) were purchased from Cayman Chemical Company (Ann Arbor, MI, USA) and FerroOrange (Cat. No. F374) from Dojindo Molecular Technologies Inc. USA. Primary antibodies used in this study included rabbit polyclonal anti‐FTH1 (Cat. No. ab75973), rabbit polyclonal anti‐4‐HNE (Cat. No. ab46545), rabbit monoclonal anti‐ACSL4/FACL4 (Cat. No. ab205199), and rabbit monoclonal anti‐xCT (Cat. No. ab307601), all of which were purchased from Abcam (Boston, MA, USA). The mouse monoclonal anti‐GPX4 (Cat. No. sc‐166570) and the anti‐β‐actin‐HRP antibody (Cat. No. sc‐47778 HRP) were obtained from Santa Cruz Biotechnology (Dallas, TX, USA). Secondary antibodies, including Peroxidase‐AffiniPure Goat Anti‐Rabbit IgG (H + L) (Cat. No. 111‐035‐003) and Peroxidase‐AffiniPure Goat Anti‐Mouse IgG (H + L) (Cat. No. 115‐035‐003) were purchased from Jackson ImmunoResearch (West Grove, PA, USA).

### 2.2 Cell Culture and Treatments

BV2 murine microglial cells (Gifted by Dr. Sanjay Maggirwar, University of Rochester Medical Center, Rochester, NY, USA) were maintained in high‐glucose Dulbecco's modified Eagle's medium (DMEM) supplemented with 10% fetal bovine serum (FBS) and 1% penicillin–streptomycin (1 mL/100 mL). Cultures were grown under standard humid conditions (37°C, 5% CO_2_, 95% air) and subcultured at 80% confluence. For experimental assays, cells were seeded at a density of 0.1 × 10^6^ cells/well in six‐well plates and serum‐starved overnight to synchronize the cell population. To assess HIV Tat‐mediated ferroptotic signaling, BV2 cells were exposed to recombinant HIV Tat (100 ng/mL, 48 h) in serum‐free medium. In parallel, the protective role of H_2_S was evaluated by pretreating cells with the H_2_S donor NaHS (100 μM, 1 h) prior to HIV Tat exposure. Medium was replenished every 24 h to maintain reagent stability and nutrient balance. LPS (0127: B8, Sigma‐Aldrich, 100 ng/mL) was used as a positive control for the induction of proinflammatory cytokines. TBHP (tert‐butyl hydrogen peroxide, 1 μM for 2 h) was used as a positive control for ROS generation and was provided with kit (Cat. No. I3600) procured from Thermo Fisher Scientific Inc., Eugene, OR, USA.

### 2.3 Western Blotting

Protein expression analysis was performed using western blotting procedures optimized previously in the laboratory (32542594). Following the designated treatments, cells were rinsed twice with ice‐cold phosphate‐buffered saline (PBS) and lysed in RIPA buffer supplemented with protease and phosphatase inhibitors. Lysates were incubated on ice for 30 min and subsequently sonicated with brief 80 Hz pulses to ensure complete disruption of cellular membranes. After centrifugation at 12,000 × g for 15 min at 4°C, the supernatant was collected, and protein concentration was quantified using the bicinchoninic acid (BCA) assay (Thermo Fisher Scientific, Waltham, MA, USA, Cat No. 23227). Equal amounts of protein were resolved on 10%–15% sodium dodecyl sulfate‐polyacrylamide gel electrophoresis (SDS‐PAGE) and transferred onto polyvinylidene difluoride (PVDF) membranes (Millipore Sigma, MO, USA, Cat No. IPVH00010) at 100 V for 90 min under cold conditions. The membranes were then blocked in 5% nonfat milk for 2 h at room temperature, followed by overnight incubation at 4°C with primary antibodies targeting ACSL4, GPX4, SLC7A11, FTH1, and 4‐HNE. After three washes in Tris‐buffered saline with Tween‐20 (TBST) buffer, membranes were probed with Horseradish peroxidase (HRP)‐conjugated secondary antibodies for 2 h at room temperature. Protein bands were visualized using an enhanced chemiluminescent substrate and imaged with a FluorChem M system (Cell Biosciences, Santa Clara, CA, USA). β‐actin served as the loading control. Band intensities were quantified using NIH ImageJ software [30].

### 2.4 Immunofluorescence Microscopy

Immunofluorescence staining was performed to visualize protein localization and expression changes of key ferroptosis markers. BV2 cells were plated on sterile glass coverslips (0.05 × 10^6^ cells/well) in 24‐well plates and treated as indicated. Cells were fixed with 4% paraformaldehyde (pH 7.2) for 15 min at room temperature and permeabilized with 0.1% Triton X‐100 for 10 min. After blocking with 5% normal goat serum in PBS containing 0.1% Triton X‐100 for 2 h, cells were incubated overnight at 4°C with primary antibodies against GPX4 and SLC7A11. The following day, cells were washed and incubated for 1 h at room temperature with fluorophore‐conjugated secondary antibodies, counterstained with DAPI, and mounted using antifade reagent. Images were captured using a Zeiss Axio Observer Z1 inverted fluorescence microscope (Carl Zeiss Microscopy, White Plains, NY, USA) equipped with AxioVs 40 Version 4.8.0.0 software (Carl Zeiss MicroImaging GmbH). Quantitative fluorescence analysis was performed from at least five random fields per condition and analyzed by ZEN2.3 blue edition.

### 2.5 C11‐BODIPY 581/591 Imaging

To determine HIV Tat‐induced lipid peroxidation and its modulation by NaHS, BV2 cells were treated with HIV Tat (100 ng/mL), NaHS (100 μM), RSL3 (500 nM), or Erastin (25 μM) for 48 h. One hour prior to assay termination, cells were incubated with 2 μM C11‐BODIPY 581/591 for 30 min at 37°C, followed by 1 μM Hoechst (Thermo Fisher Scientific, Waltham, MA, USA, Cat No. 33342) nuclear counterstaining for the last 5 min. After washing with Hank's Balanced Salt Solution (HBSS), live‐cell fluorescence images were acquired using the Zeiss Z1 inverted microscope. The shift in fluorescence emission from red (reduced) to green (oxidized) was quantified as an index of lipid peroxidation analyzed by ZEN2.3 blue edition.

### 2.6 Intracellular ROS Quantification

Intracellular ROS generation was measured using the redox‐sensitive fluorescent probe. Following treatment, cells were incubated with 10 μM DCFH_2_‐DA for 30 min at room temperature in the dark, followed by 1 μM Hoechst staining for 5 min. After washing thrice with PBS, cells were maintained in calcium‐ and magnesium‐free HBSS for immediate live‐cell imaging using inverted fluorescence microscope (Carl Zeiss Microscopy, White Plains, NY, USA) equipped with AxioVs 40 Version 4.8.0.0 software (Carl Zeiss MicroImaging GmbH). Relative fluorescence intensity was analyzed by ZEN2.3 blue edition.

### 2.7 Determination of the Labile Iron Pool (LIP)

To quantify cytosolic Fe^2+^ levels, both fluorescence‐based and biochemical assays were performed. For the fluorescence assay, treated BV2 cells grown on coverslips were incubated with 1 μM FerroOrange and 1 μM Hoechst for 5 min at 37°C. After gentle washing, fluorescence images were captured using inverted fluorescence microscope (Carl Zeiss Microscopy, White Plains, NY, USA) equipped with AxioVs 40 Version 4.8.0.0 software (Carl Zeiss MicroImaging GmbH), and mean fluorescence intensity was calculated from multiple fields and analyzed by ZEN2.3 blue edition. For biochemical quantification, cells were lysed in the assay buffer supplied with the Iron Assay Kit.

### 2.8 Quantification of Proinflammatory Cytokines by qPCR

Total RNA was isolated and reverse‐transcribed using the Verso cDNA Synthesis Kit (Cat. No. AB‐1453/B; Thermo Fisher Scientific, Pittsburgh, PA, USA). Gene expression levels of the proinflammatory cytokines TNFα, IL‐1β, and IL‐6 were quantified using SYBR Green probes (Applied Biosystems, Grand Island, NY, USA). qPCR reactions were carried out on a QuantStudio 3 Real‐Time PCR System (Applied Biosystems), which provided precise amplification and real‐time fluorescence quantification. Expression levels were normalized to GAPDH, and relative quantification was performed using the comparative Ct method, with fold changes expressed as 2^−ΔΔ*C*^_t_. The probe catalog number and the sequences used were: TNFα (Forward: CAGCCTCTTCTCCTTCCTGAT and Reverse: GCCAGAGGGCTGATTAGAGA), IL‐1β (Forward: CACTACAGGCTCCGAGATGA, Reverse: TCTGTCCATTGAGGTGGAGA), IL‐6 (Forward: TAGTCCTTCCTACCCCAATTTCC, Reverse: TTGGTCCTTAGCCACTCCTTC), and GAPDH (Forward: GGTGAAGGTCGGTGTGAACG, Reverse: CTCGCTCCTGGAAGATGGTG) supplied by Invitrogen (Cat. No. 10336022).

### 2.9 LDH Assay

HIV Tat‐induced cytotoxicity was evaluated by measuring LDH release into the culture medium using a commercial LDH Activity Assay Kit. Briefly, 10 μL of conditioned medium from each treatment group was mixed with 100 μL of reaction buffer containing LDH substrate. After incubation for 30 min at room temperature, absorbance was measured at 593 nm on a microplate reader. The percentage of cytotoxicity was calculated using the following formula:

$$\begin{array}{c} \text{Cytotoxicity}\;\left(\%\right)\\ =\left(\text{Test sample}-\mathrm{Low}\;\text{control}\right)/\left(\text{High control}-\mathrm{Low}\;\text{control}\right)\times 100 \end{array}$$

### 2.10 Statistical Analysis

All experiments were performed in six independent biological replicates (*n* = 6). Data are presented as mean ± standard error of the mean (SEM). Statistical analyses were conducted using GraphPad Prism (v10.0). One‐way analysis of variance (ANOVA) followed by Šídák's post hoc test was applied for multiple comparisons. *p* < 0.05 was considered statistically significant.

## 3 Results

### 3.1 NaHS Pretreatment Attenuates HIV Tat‐Induced Iron Dysregulation in Microglial Cells

**FIGURE 1:**
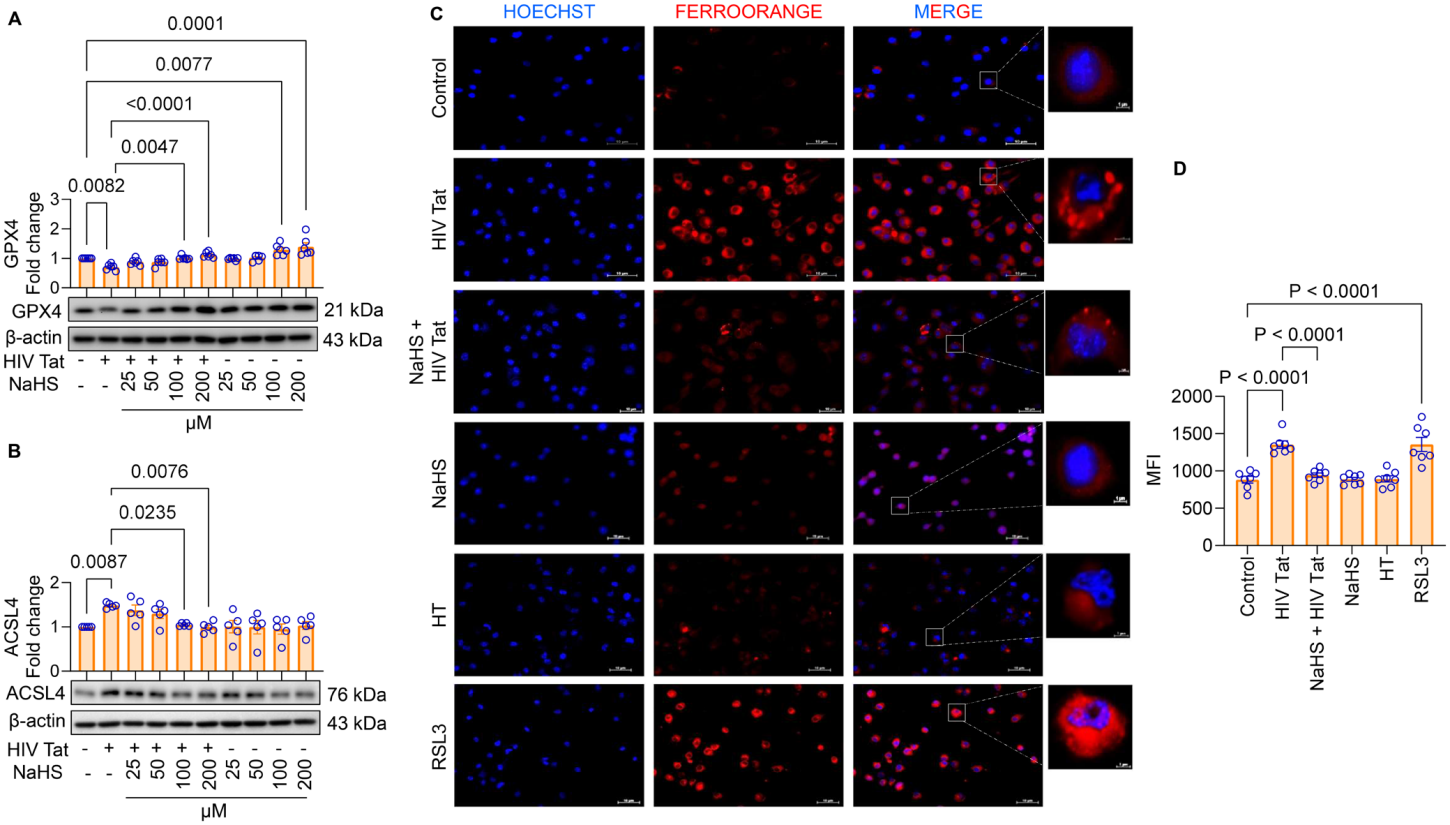
NaHS displays ferroptotic rescue potency in dose dependent manner and blocks HIV Tat‐mediated intracellular Iron levels in BV2 microglial cells. (A, B) Representative immunoblots showing the expression levels of (A) GPX4 and (B) ACSL4, along with their respective quantifications, in BV2 cells treated with various concentrations of NaHS (μM), followed by exposure to HIV Tat (100 ng/mL). (C) Representative live cell fluorescent images (10 μm, scale bar) of HIV Tat (100 ng/mL) and NaHS (100 μM) treated BV2 cells after labelling labile iron (Fe^2+^) with FerroOrange. The Hoechst staining was used to label the nucleus in blue. (D) Quantification of the mean intensity of FerroOrange was calculated for the total area per image analyzed by ZEN2.3 blue edition. The data are presented as mean ± SEM from three independent experiments, and *p* < 0.05 was considered significant. One‐way ANOVA followed by Šídák's post hoc test was used to determine the statistical significance of multiple groups.

To determine whether hydrogen sulfide modulated HIV Tat‐induced iron imbalance, BV2 microglial cells were exposed to HIV Tat (100 ng/mL) in the presence or absence of varying doses of NaHS pretreatment (100 μM, 1 h). The optimal concentration of HIV Tat (100 ng/mL) was selected based on our previous study [16]. As shown in Figure 1A,B, NaHS pretreatment dose‐dependently restored HIV Tat‐mediated dysregulated expression of iron‐handling proteins such as GPX4 and ACSL4. NaHS showed a dose‐dependent effect in the HIV Tat fixed condition; however, 100 μM concentration was the lowest effective dose that showed significance. Next, we wanted to assess the effect of HIV Tat on the cytosolic labile ferrous iron. Using the fluorescent probe FerroOrange, that binds to the free Fe^2+^ iron, we demonstrated HIV Tat‐mediated elevation of cytosolic labile Fe^2+^ levels compared with untreated control cells (Figure 1C). Pretreatment with NaHS markedly reduced HIV Tat‐mediated Fe^2+^ accumulation, indicating partial restoration of iron homeostasis. Quantification of intracellular Fe^2+^ using a specific probe further confirmed HIV Tat significantly increased the labile iron pool (LIP), whereas NaHS pretreatment normalized the iron levels to near‐control values (Figure 1D). The ferroptosis inducer RSL3 (500 nM) served as a positive control and similarly elevated the LIP, whereas heat‐inactivated Tat (HT) failed to alter iron content. Collectively, these findings demonstrated that NaHS effectively mitigated HIV Tat‐mediated iron dysregulation in microglial cells.

### 3.2 NaHS Pretreatment Restored Redox Homeostasis in HIV Tat‐Exposed Microglial Cells

**FIGURE 2:**
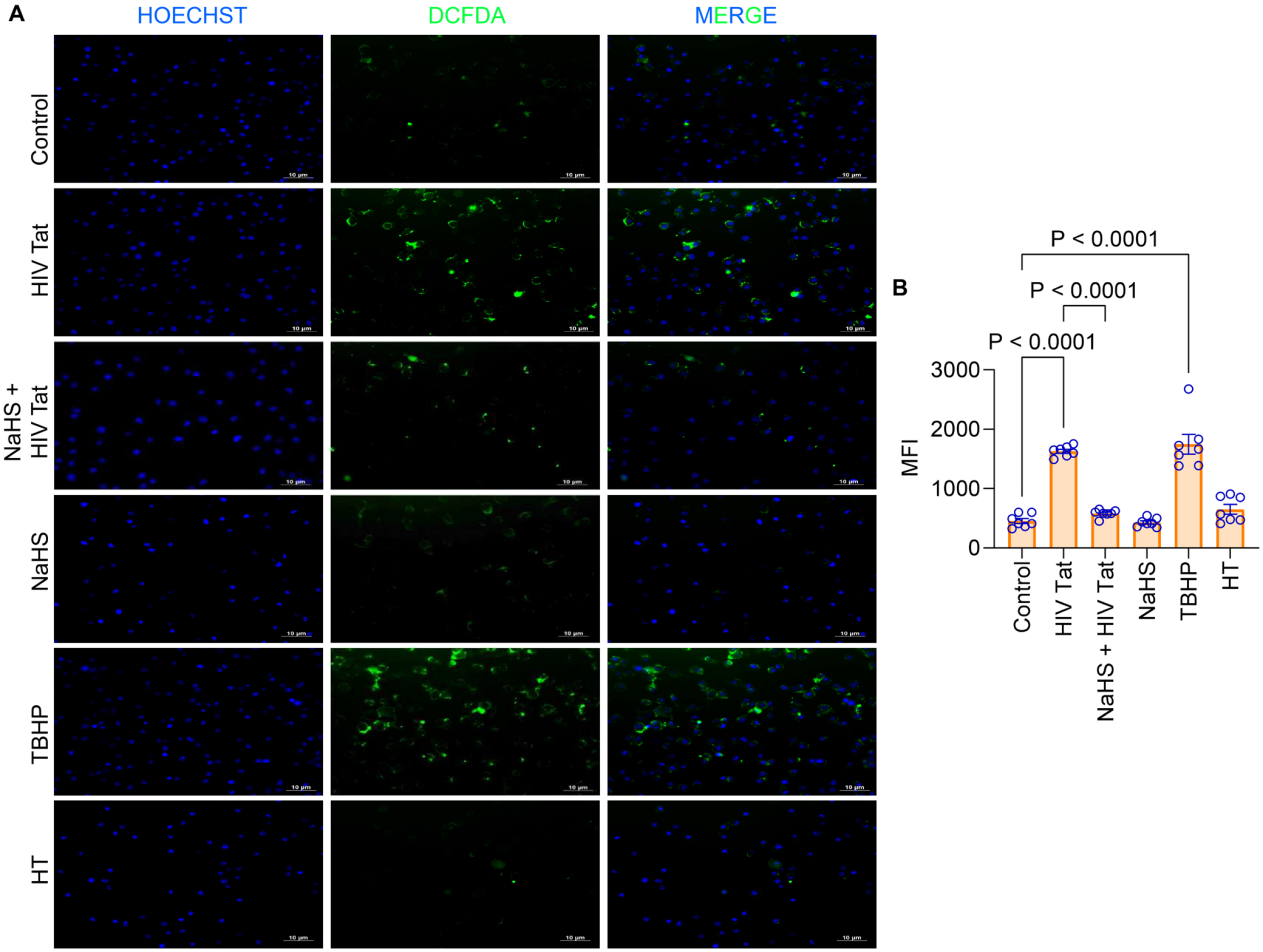
Restoration of redox homeostasis by NaHS in BV2 microglial cells exposed to HIV Tat. (A) Fluorescence microscopy (10 μm, scale bar) analysis showed that NaHS (100 μM) pretreatment reduced ROS fluorescence in HIV Tat (100 ng/mL) BV2 cells after staining with 10 μM DCFDA dye for 30 min. (B) Quantification of the mean intensity of DCFDA green fluorescence analyzed by ZEN2.3 blue edition. The data are presented as mean ± SEM from three independent experiments, and *p* < 0.05 was considered significant. One‐way ANOVA followed by Šídák's post hoc test was used to determine the statistical significance of multiple groups.

Given that excessive Fe^2+^ can catalyze the Fenton reaction and generate ROS, we next assessed whether NaHS pretreatment could mitigate HIV Tat‐induced oxidative stress. BV2 cells exposed to HIV Tat exhibited a pronounced increase in ROS fluorescence intensity as detected by the DCFH_2_‐DA probe (Figure 2A,B). Pretreatment with NaHS (100 μM) markedly suppressed HIV Tat‐induced ROS accumulation, thus restoring the intracellular redox balance. Positive control tert‐butyl hydroperoxide (TBHP) strongly upregulated ROS generation, thus confirming assay sensitivity, whereas HT elicited no measurable effect. These findings underscored the role of NaHS in maintaining the redox equilibrium in HIV Tat‐exposed microglia by curbing excessive ROS formation.

### 3.3 NaHS Pretreatment Reduced Lipid Peroxidation in HIV Tat‐Exposed Microglial Cells

**FIGURE 3:**
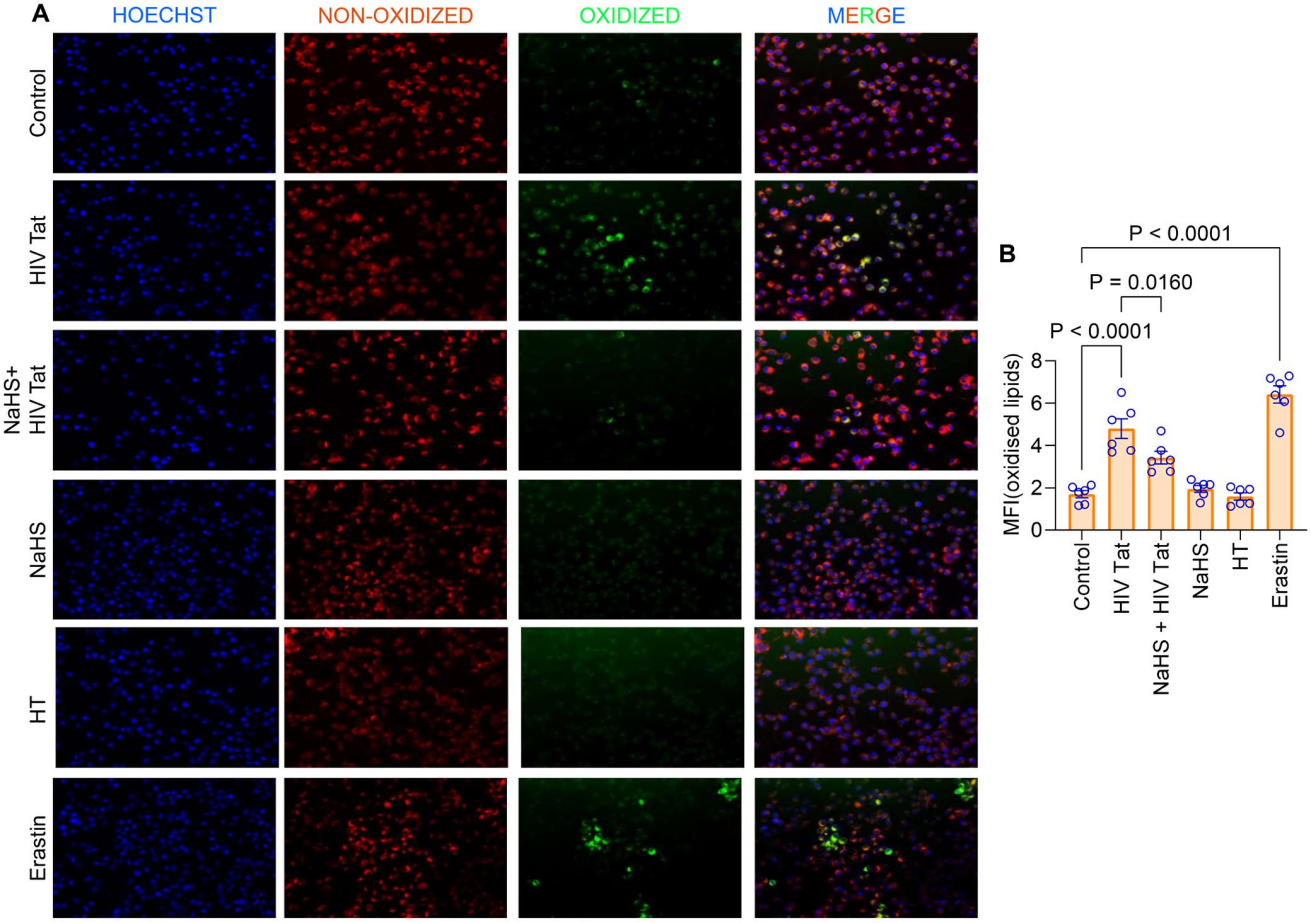
NaHS attenuates lipid peroxidation in BV2 microglial cells exposed to HIV Tat. (A) Representative fluorescent microscopy images showing the oxidized lipid levels in HIV Tat (100 ng/mL) exposed BV2 microglial cells and NaHS pretreatment (100 μM; 1 h) in HIV‐ Tat (100 ng/mL) exposed BV2; magnification: ×20 and scale bar, 10 μm. (B) Quantification shows the shift in C11‐BODIPY to green, which depicts the oxidized lipids and their mean fluorescence was analyzed by ZEN2.3 blue edition. The data are presented as mean ± SEM from three independent experiments, and *p* < 0.05 was considered significant. One‐way ANOVA followed by Šídák's post hoc test was used to determine the statistical significance of multiple groups.

Since lipid peroxidation is a central hallmark of ferroptotic cell death, we next investigated whether NaHS pretreatment could reverse HIV Tat‐induced oxidative membrane damage. Using live‐cell imaging with the redox‐sensitive fluorescent probe C11‐BODIPY 581/591, HIV Tat‐exposed BV2 cells displayed a pronounced shift from red to green fluorescence, indicative of increased lipid oxidation (Figure 3A). Similarly, treatment with the ferroptosis inducer Erastin (2.5 μM) resulted in comparable lipid peroxidation. In contrast, NaHS pretreatment (100 μM) substantially downregulated the oxidized lipid signal, restoring the red fluorescence typical of non‐oxidized membranes (Figure 3B). Quantitative analysis confirmed that NaHS pretreatment significantly reduced HIV Tat‐induced membrane lipid oxidation. These results demonstrated that NaHS effectively counteracted HIV Tat‐driven lipid peroxidation and preserved membrane integrity of microglia.

### 3.4 NaHS Mitigated HIV Tat‐Induced Ferroptosis by Modulating Ferroptotic Protein Expressions

**FIGURE 4:**
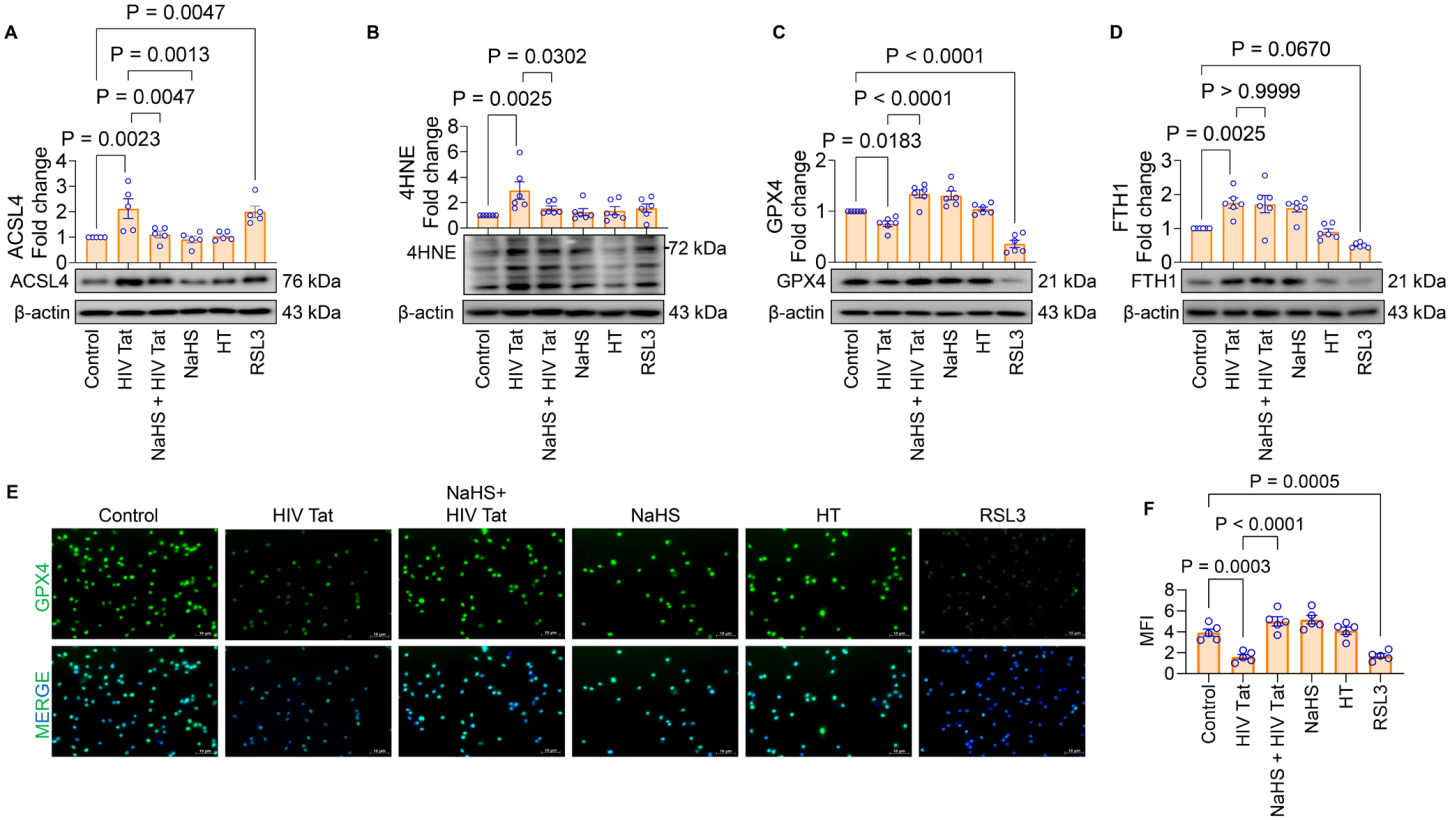
Modulation of ferroptotic markers by NaHS mitigates HIV Tat‐induced ferroptosis in BV2 microglial cells. Representative immunoblots showing the expression levels of (A) ACSL4, (B) 4‐HNE, (C) GPX4, and (D) FTH1 with their respective quantifications in NaHS (100 μM; 1 h) pretreated and HIV Tat (100 ng/mL) exposed BV2 cells. (E) Representative fluorescence images of GPX4 and (F) quantification of mean fluorescence in NaHS (100 μM; 1 h) pretreated and HIV Tat (100 ng/mL) and 0.5 μM RSL3 exposed BV2 cells. The data are presented as mean ± SEM from *n* = 6 independent experiments, and *p* < 0.05 was considered significant. One‐way ANOVA followed by Šídák's post hoc test was used to determine the statistical significance of multiple groups.

To further elucidate whether NaHS directly influenced ferroptotic signaling, we examined the expression of key ferroptosis‐related proteins. Western blot analysis revealed that HIV Tat exposure markedly increased the levels of the pro‐ferroptotic enzyme, ACSL4, and the lipid peroxidation product, 4‐HNE, concomitant with a decrease in the antioxidant enzyme, GPX4 (Figure 4A–C). Pretreatment with NaHS significantly attenuated ACSL4 and 4‐HNE expression while restoring GPX4 levels to those comparable with control cells. Expression of FTH1, which reflects cellular iron storage, remained unchanged in HIV Tat‐exposed BV2 microglial cells (Figure 4D). Cells exposed to the ferroptosis inducer RSL3 exhibited the expected increase in ferroptotic markers, validating assay reliability. Further, as shown in Figure 4E,F, immunofluorescence analysis showed significantly higher expression of GPX4 levels in NaHS pretreated conditions compared to HIV Tat‐exposed BV2 cells. Together, these findings establish that NaHS suppresses HIV Tat‐induced ferroptosis by simultaneously downregulating pro‐ferroptotic mediators and reinforcing the GPX4‐dependent antioxidant defense.

### 3.5 NaHS Activated the SLC7A11‐GPX4 Axis to Confer Tat‐Mediated Anti‐Ferroptotic Protection

**FIGURE 5:**
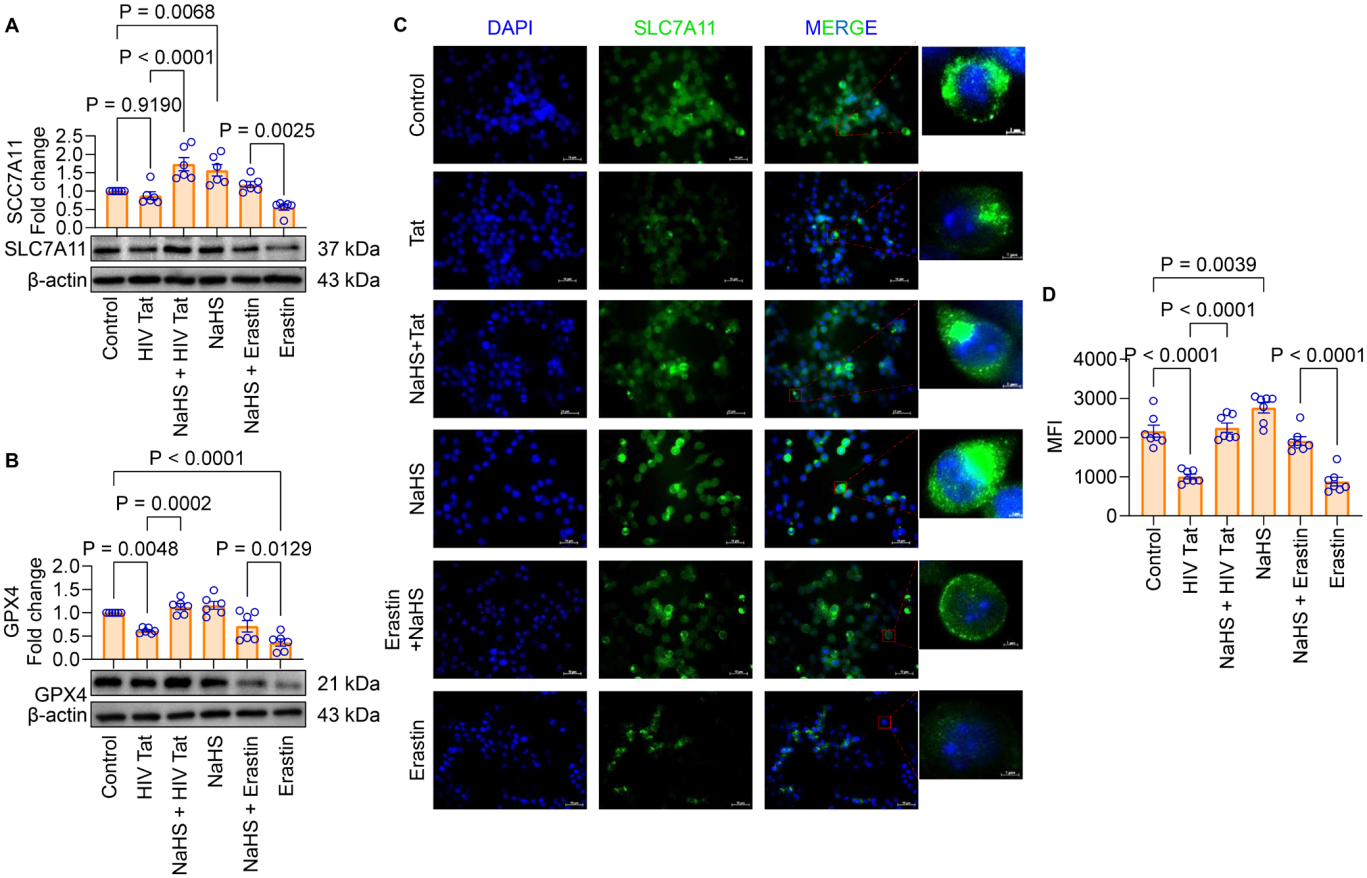
NaHS‐induced activation of the SLC7A11‐GPX4 pathway protects against HIV Tat‐mediated ferroptosis in BV2 microglial cells. Representative immunoblots showing the expression levels of (A) SLC7A11, (B) GPX4, with their respective quantifications of NaHS (100 μM; 1 h) pretreated, followed by HIV Tat (100 ng/mL) and erastin (2.5 μM) exposed BV2 cells. (C) Representative fluorescence images of SLC7A11 in the presence of HIV Tat (100 ng/mL), NaHS (100 μM), and Erastin (2.5 μM), and (D) Quantification of mean fluorescence of respective treatments analyzed by ZEN2.3 blue edition. The data are presented as mean ± SEM from *n* = 6 independent experiments, and *p* < 0.05 was considered significant. One‐way ANOVA followed by Šídák's post hoc test was used to determine the statistical significance of multiple groups.

Since GPX4 activity is closely linked to its upstream regulator SLC7A11, we next evaluated whether NaHS pretreatment could modulate the SLC7A11‐GPX4 axis. As expected, both HIV Tat and erastin (2.5 μM, a pharmacological inhibitor of SLC7A11) exposure led to a significant reduction in the expression of SLC7A11 (Figure 5A). Remarkably, NaHS pretreatment restored SLC7A11 levels in both HIV Tat‐ and erastin‐treated microglia, and this effect was paralleled by upregulated expression of GPX4 (Figure 5B). To further validate these findings, we performed immunofluorescence imaging and observed that SLC7A11 expression was markedly reduced in the presence of HIV Tat and erastin compared with control cells. Notably, NaHS pretreatment in HIV Tat‐ and erastin‐exposed BV2 cells restored the expression of SLC7A11, as shown in Figure 5C,D. These results collectively indicate that activation of the SLC7A11‐GPX4 axis underlies the anti‐ferroptotic action of NaHS in HIV Tat‐exposed microglia.

### 3.6 NaHS Pretreatment Attenuates HIV Tat‐Induced Proinflammatory Cytokine Expression, Microglial Activation, and Cytotoxicity

**FIGURE 6:**
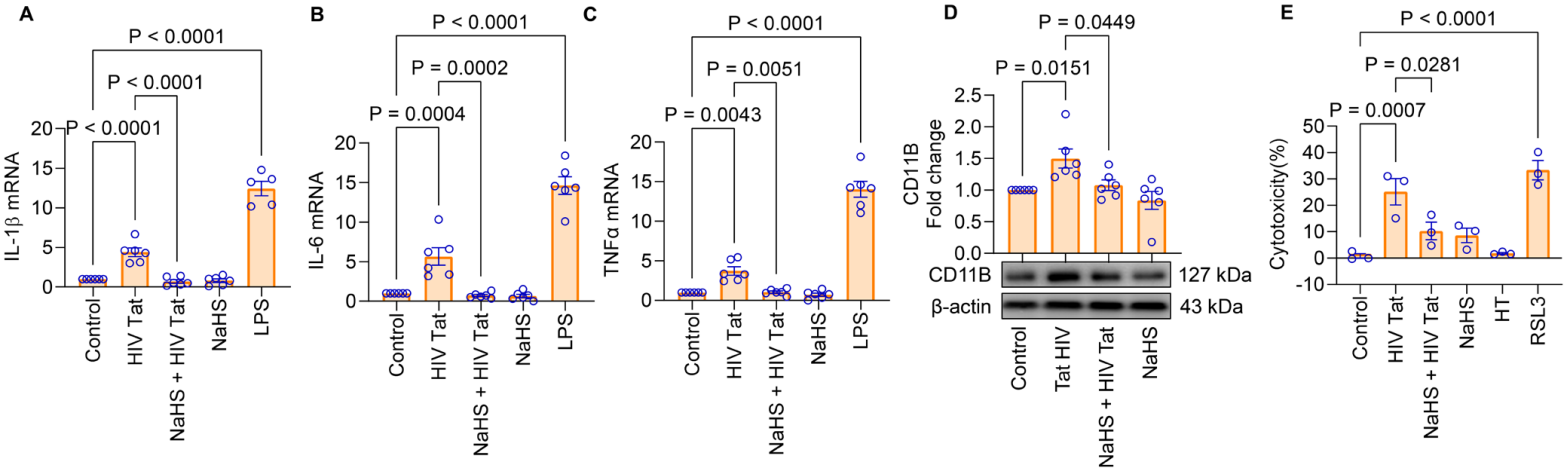
Inhibition of HIV Tat‐mediated inflammation and toxicity by NaHS pretreatment in BV2 microglial cells. Bar graphs showing the expression levels of proinflammatory cytokines such as (A) IL1β, (B) IL6, and (C) TNFα in NaHS (100 μM; 1 h) pretreated HIV Tat (100 ng/mL) and LPS (100 μM) exposed BV2 cells. (D) Representatives immunoblot of CD11B with its quantification depicting the microglial activation in NaHS (100 μM; 1 h) pretreated and HIV Tat (100 ng/mL) exposed BV2 cells. (E) Representative bar graph showing the percentage LDH release from BV2 cells pretreated with NaHS (100 μM; 1 h) followed by HIV Tat (100 ng/mL, 48 h) exposure, analyzed by LDH assay kit. The data are presented as mean ± SEM from *n* = 6 independent experiments, and *p* < 0.05 was considered significant. One‐way ANOVA followed by Šídák's post hoc test was used to determine the statistical significance of multiple groups.

Given its protective effects on HIV Tat‐induced dysregulation of iron homeostasis, we next evaluated whether NaHS could suppress proinflammatory cytokine expression, microglial activation, and cytotoxicity in response to HIV Tat. BV2 cells were pretreated with NaHS (100 μM) for 1 h, followed by exposure to HIV Tat (100 ng/mL) for 48 h. The mRNA levels of key proinflammatory cytokines, including IL‐1β, IL‐6, and TNFα, were quantified by qPCR. As shown in Figure 6A–C, NaHS pretreatment significantly attenuated the HIV Tat‐induced upregulation of all three cytokines. These findings suggest that the NaHS‐mediated inhibition of ferroptosis effectively dampens HIV Tat‐driven inflammatory signaling in BV2 microglial cells.

To further assess its cell protective effects, we investigated whether NaHS could also mitigate microglial activation. BV2 cells were pretreated with NaHS (100 μM) and subsequently exposed to HIV Tat (100 ng/mL) for 48 h. Cell lysates were analyzed for expression of the microglial activation marker CD11b. As shown in Figure 6D, NaHS pretreatment markedly reduced HIV Tat‐induced CD11b expression, indicating that its protective actions extend to suppressing microglial activation. Finally, to determine whether the anti‐ferroptotic effects of NaHS translated into improved cell viability, cytotoxicity was assessed using the LDH release assay. HIV Tat exposure resulted in a significant increase in extracellular LDH activity, indicative of membrane damage and cell death (Figure 6E). In contrast, NaHS pretreatment substantially reduced LDH release, demonstrating its cytoprotective potential. Together, these results corroborate that NaHS alleviates HIV Tat‐induced microglial cytotoxicity by preventing ferroptotic membrane disruption.

## 4 Discussion

Ferroptosis, a regulated form of cell death driven by iron‐dependent lipid peroxidation, has recently emerged as a pivotal mechanism in neurodegenerative and neuroinflammatory disorders. Mounting evidence suggests that the HIV regulatory protein, Tat, contributes to neuronal injury by promoting oxidative stress, mitochondrial dysfunction, and glial activation within the CNS [31, 32]. Our previous studies and those of others have demonstrated that HIV Tat dysregulates microglial homeostasis, thereby facilitating chronic neuroinflammation and neuronal toxicity in people living with HIV [16, 33, 34, 35, 36]. Notably, time‐course analyses from our prior work suggest that the induction of inflammatory mediators may precede the emergence of ferroptosis‐associated signaling [16, 37]. The present work expands on this understanding by identifying H_2_S as a potent endogenous modulator capable of mitigating HIV Tat‐induced ferroptosis in microglial cells.

Microglia serve as the principal immune effector cells of the CNS and play a vital role in both neuroprotection and neurotoxicity [38]. Persistent HIV Tat exposure has been shown to reprogram microglia toward a pro‐oxidant phenotype characterized by excessive ROS generation and proinflammatory cytokines release [4]. Here, we confirmed that HIV Tat triggers ferroptosis in BV2 microglia, as evidenced by iron accumulation, lipid peroxidation, and upregulation of the canonical ferroptosis marker ACSL4. Consistent with previous observations in postmortem HIV‐infected brains and HIV transgenic rodent models, HIV Tat‐induced increases in the LIP correlated with diminished expression of the antioxidant enzyme GPX4, a master regulator that detoxifies lipid peroxides through glutathione utilization [16]. These findings underscore ferroptosis as a mechanistic link between HIV Tat toxicity, oxidative stress, and neuroinflammation.

H_2_S has gained recognition as a vital gaseous signaling molecule, alongside nitric oxide and carbon monoxide, and exerts broad cytoprotective effects in the nervous and cardiovascular systems [23, 25, 26, 27]. Notably, several reports have indicated that endogenous H_2_S levels are reduced in HIV infection and associated comorbidities, leading to dysregulated redox signaling [20]. In the current study, supplementation with the H_2_S donor NaHS effectively restored redox equilibrium in HIV Tat‐exposed microglia. NaHS pretreatment markedly attenuated HIV Tat‐induced Fe^2+^ accumulation, curtailed ROS production, and prevented oxidative membrane damage, demonstrating that H_2_S restores iron and redox balance. This is consistent with emerging evidence that H_2_S regulates iron metabolism by promoting ferritin expression and limiting iron‐catalyzed Fenton chemistry [39, 40]. A central finding of this study is that NaHS upregulates the SLC7A11‐GPX4 antioxidant defense axis. HIV Tat exposure and the pharmacological inhibitor, erastin, both reduced SLC7A11 expression, thereby limiting cystine uptake and glutathione synthesis. In contrast, NaHS pretreatment restored SLC7A11 levels and increased GPX4 expression, effectively counteracting lipid peroxidation and cell death. This observation is consistent with reports that H_2_S enhances glutathione biosynthesis and preserves GPX4 activity by maintaining cysteine availability [26, 41]. The concurrent downregulation of ACSL4 and suppression of 4‐HNE further confirm that NaHS modulates the ferroptotic cascade at multiple checkpoints, including iron accumulation, lipid peroxidation, and antioxidant impairment, thereby providing comprehensive protection against HIV Tat‐induced oxidative injury. Notably, NaHS did not significantly alter FTH1 levels in HIV Tat‐exposed BV2 microglia, even though other inflammatory and iron‐related markers were modulated.

Beyond ferroptosis, HIV Tat‐stimulated microglia release proinflammatory mediators that contribute to the broader neuroinflammatory milieu of NeuroHIV [16]. Our findings also showed that NaHS treatment reduced HIV Tat‐induced cytotoxicity, as measured by LDH release, consistent with its ability to inhibit ferroptosis. Moreover, NaHS markedly decreased the HIV Tat‐induced expression of the proinflammatory cytokines IL‐1β, IL‐6, and TNF‐α, indicating a broader anti‐neuroinflammatory effect. The connection between lipid peroxidation and cytokine production is well established in neuroinflammation, and our results suggest a potential mechanism by which H_2_S may attenuate these inflammatory responses. Collectively, these findings highlight H_2_S as a dual‐acting modulator that limits both ferroptotic and inflammatory signaling pathways in HIV Tat‐exposed microglia.

The current study defines a mechanistic framework in which HIV Tat induces ferroptosis in microglia through excessive iron accumulation, ROS generation, lipid peroxidation, and suppression of the SLC7A11‐GPX4 antioxidant axis. Restoration of H_2_S bioavailability via NaHS supplementation effectively counteracted these pathogenic events by modulating iron homeostasis, preserving redox balance, and reinforcing antioxidant defenses, thereby maintaining microglial viability and functional integrity. These findings provide a strong rationale for considering H_2_S donors or H_2_S‐releasing compounds as adjunctive therapeutic strategies that warrant further validation for NeuroHIV, particularly in combination with cART regimens that fail to address HIV Tat‐mediated neurotoxicity and chronic neuroinflammation.

Several limitations of the present study warrant consideration. First, the experimental design relied on exposure to a single HIV protein (Tat), which does not fully capture the complexity of viral protein interactions encountered during HIV infection. Second, the study was conducted exclusively in microglial in vitro models and did not incorporate neuron–glia co‐culture systems or in vivo paradigms that more accurately recapitulate the neuroimmune environment of the CNS. Future studies employing in vivo HIV transgenic or SIV models will therefore be essential to validate these protective mechanisms and to determine the long‐term impact of H_2_S supplementation on neuroinflammatory and neurodegenerative outcomes. Importantly, future investigations will aim to mechanistically define the role of the H_2_S‐SLC7A11‐GPX4 axis in HIV‐associated neuroinflammation using microglia‐specific SLC7A11 and GPX4 conditional knockout HIV mouse models. This genetic approach will be critical for distinguishing bona fide anti‐ferroptotic mechanisms from potential off‐target effects inherent to pharmacological interventions and for establishing causality between H_2_S signaling and ferroptosis suppression in NeuroHIV.

In summary, our study provides compelling evidence that H_2_S protects microglial cells from HIV Tat‐mediated ferroptosis by modulating iron and redox balance, downregulating ACSL4 and 4‐HNE, and activating the SLC7A11‐GPX4 antioxidant axis. This dual regulation of pro‐ and anti‐ferroptotic mechanisms confers significant cytoprotection and may attenuate microglial‐driven neuroinflammation in NeuroHIV. Targeting ferroptosis with H_2_S donors thus could be a helpful and promising strategy for managing oxidative and neuroinflammation associated with NeuroHIV.

## Author Contributions

**Aitizaz Ul Ahsan:** writing – original draft, writing – review and editing, investigation, validation, visualization, formal analysis, methodology, data curation. **Frida L. Martínez‐Cuevas:** writing – review and editing, formal analysis, data curation. **Elias Horanieh:** writing – review and editing, visualization. **Seema Singh:** writing – review and editing, validation. **Uma Maheswari Deshetty:** writing – review and editing, validation. **Palsamy Periyasamy:** conceptualization, writing – review and editing, funding acquisition, resources, supervision. **Shilpa Buch:** conceptualization, writing – review and editing, funding acquisition, resources, supervision.

## Funding

This work was supported by startup funding from the UNMC.

## Ethics Statement

This study did not involve human participants or experimental animals. All experiments were conducted exclusively using the established mouse microglial BV2 cell line. As such, approval from an Institutional Review Board (IRB) or Institutional Animal Care and Use Committee (IACUC) was not required, and no ethical approval was applicable for this work.

## Conflicts of Interest

The authors declare no conflicts of interest.

## Data Availability

The data that support the findings of this study are available from the corresponding author upon reasonable request.
